# IDENTIFYING PREDICTORS OF GUILT FOLLOWING PLACEMENT OF FAMILY MEMBER WITH DEMENTIA INTO RESIDENTIAL LONG-TERM CARE

**DOI:** 10.1093/geroni/igz038.1806

**Published:** 2019-11-08

**Authors:** Tamara L Statz, Colleen M Peterson, Robyn Birkeland, Aneri Shah, Kobe Perez, Joseph E Gaugler

**Affiliations:** University of Minnesota - School of Public Health, Division of Health Policy and Management, Minneapolis, Minnesota, United States

## Abstract

Family caregivers of persons with dementia experience guilt for various reasons when placing their family member into residential long-term care (RLTC). Research has shown a relationship between guilt and overall caregiver burden; however, literature on predictors of guilt related to caregiving following RLTC placement is limited. The Residential Care Transition Module (RCTM) provides counseling and psychoeducation to family caregivers who have recently moved their family member with Alzheimer’s disease or a related dementias into RLTC. This semi-structured intervention provides counseling on various topics including guilt, grief, and family dynamics. Using treatment group data (N=87) from the parent RCTM randomized controlled trial, we identified the impact of caregiver status (i.e., adult child vs spousal caregivers), sense of caregiver competence, and relationship closeness on baseline measures of guilt status and magnitude. Preliminary analyses showed that adult child status was associated with greater prevalence of guilt (37.5%) compared to spousal caregivers (26.7%). In addition, qualitative case notes were coded to identify common themes of experiences of guilt to inform practice. Examples include: Guilt related to no longer being able to care for the family member at home; not meeting self-dictated expectations (e.g., not visiting or staying long enough); and feeling guilted into being a caregiver in the first place. The current analyses aim to help practitioners better predict risk for placement-related guilt and highlight specific issues practitioners should consider to help mitigate such feelings. Specific opportunities for intervention are discussed.

